# Association between white matter alterations on diffusion tensor imaging and incidence of frailty in older adults with cardiometabolic diseases

**DOI:** 10.3389/fnagi.2022.912972

**Published:** 2022-07-27

**Authors:** Yoshiaki Tamura, Keigo Shimoji, Joji Ishikawa, Yuji Murao, Fumino Yorikawa, Remi Kodera, Kazuhito Oba, Kenji Toyoshima, Yuko Chiba, Aya M. Tokumaru, Atsushi Araki

**Affiliations:** ^1^Department of Diabetes, Metabolism, and Endocrinology, Tokyo Metropolitan Geriatric Hospital, Tokyo, Japan; ^2^The Center for Comprehensive Care and Research for Prefrailty, Tokyo Metropolitan Geriatric Hospital, Tokyo, Japan; ^3^Department of Diagnostic Radiology, Tokyo Metropolitan Geriatric Hospital, Tokyo, Japan; ^4^Department of Cardiology, Tokyo Metropolitan Geriatric Hospital, Tokyo, Japan

**Keywords:** diabetes mellitus, diffusion tensor imaging, frailty, older adults, white matter alteration

## Abstract

Diffusion tensor imaging (DTI) can be used for the early detection of abnormal changes in the integrity of cerebral white matter tracts, and we have previously reported that these changes are associated with indices of early atherosclerotic lesions. Although these changes have been demonstrated to be associated with the incidence of frailty in older adults, no studies have investigated this relationship in patients at high risk for vascular disease. In this longitudinal study, we followed outpatients with cardiometabolic diseases for a maximum of 6 years (median, 3 years) and evaluated the association of baseline DTI data of seven white matter tracts with the incidence of frailty. The modified version of the Cardiovascular Health Study criteria and the Kihon Checklist were used as indices of frailty; fractional anisotropy (FA) and mean diffusivity (MD) were used as indices of white matter changes. Patients who developed frailty based on both indices had low FA and high MD in many of the tracts tested, with the most significant difference found in the MD of the anterior thalamic radiation (ATR). Cox proportional hazard model analysis revealed a significantly high risk of frailty defined by both indices in the groups with high MD values in the left ATR. Similar results were found in patients with diabetes mellitus but not in those without diabetes mellitus. Therefore, abnormalities in the integrity of the left ATR could be associated with the progression of frailty in older adults with cardiometabolic disease, particularly those with diabetes mellitus.

## Introduction

The population of developed countries is currently aging significantly, thereby escalating the economic burden of medical and nursing care for older adults. Frailty is a condition in which older people become vulnerable to various stresses outside the process of aging (Fried et al., 2001). It has been established that physically frail older adults often need nursing care and have an increased death rate (Ensrud et al., 2008).

To decrease the incidence of frailty, identifying high-risk individuals and early intervention are crucial. Hence, various potential frailty markers are currently under investigation. White matter hyperintensity (WMH) detected by fluid-attenuated inversion recovery magnetic resonance imaging (MRI) reflects small vessel disease in the cerebrum; we had previously reported that the WMH volumes in the whole cerebrum or periventricular lesions are associated with impairments in instrumental activities of daily living (Tamura et al., 2017). Diffusion tensor imaging (DTI), an imaging technology that uses diffusion-weighted images in multiple dimensions, has recently been developed. It uses the alteration of the diffusion anisotropy of water molecules, which in normal tracts move along the direction of the major axis of the neuron bundles. DTI has several advantages over WMH analysis, including that it can depict white matter lesions in tracts connecting various cerebral areas, such as the frontal cortex and thalamus (Assaf and Pasternak, 2008). Moreover, slight changes in white matter tracts can be detected earlier than with the classic WMH imaging method.

Recently, we showed that changes in DTI values in some white matter tracts are associated with indices of subclinical atherosclerosis, such as the ankle-brachial index (Tamura et al., 2021a). In a cross-sectional study of the same patients, we also demonstrated that abnormalities in white matter integrity in certain tracts, such as the left anterior thalamic radiation (ATR, lATR) and right inferior fronto-occipital fasciculus (IFOF, rIFOF), are associated with sarcopenia and its component factors (Tamura et al., 2021b). To the best of our knowledge, only one cross-sectional study (Avila-Funes et al., 2017) and one longitudinal study (Maltais et al., 2020) have been conducted on the association between white matter integrity and frailty. However, the participants of both studies were community-dwelling older adults; the influence of white matter lesions on frailty among those at risk for vascular diseases is unknown.

Accumulation of cardiometabolic diseases could be a risk of frailty. In a recent report, it has been shown that as the number of cardiometabolic diseases increases [hypertension, diabetes mellitus (DM), dyslipidemia, cardiac diseases, and stroke], the prevalence of frailty also increases in parallel (Gao et al., 2021). Among these diseases, DM is an independent strong risk factor for frailty. Since it has been shown in a meta-analysis that the incidence of frailty is significantly high in patients with diabetes mellitus and those who are affected with frailty are at high risk for mortality (Hanlon et al., 2020), it is of importance to screen for frailty and appropriately intervene in these high-risk patients at its early stage. In this context, it is meaningful to investigate the association between white matter alteration and the incidence of frailty in patients with cardiometabolic diseases, especially those with DM.

This study aimed to investigate the association between changes in DTI values of white matter tracts and the incidence of frailty, defined using the modified version of the Cardiovascular Health Study (CHS) criteria (mCHS) and Kihon Checklist (KCL), in outpatients with cardiometabolic diseases including those with DM (62.5%) and further verify whether these changes could be used as an early predictive marker of frailty in these high risk population.

## Patients and Methods

### Patients

This longitudinal study analyzed data from 184 patients of a previous study (Tamura et al., 2021b). As described previously, they were outpatients aged ≥65 years at the frailty clinic and underwent brain MRI. In principle, these patients are recommended to visit the frailty clinic for an annual assessment of frailty status. Most patients were treated at the Departments of Diabetes and Cardiology and had cardiometabolic diseases, such as hypertension, diabetes mellitus, dyslipidemia, ischemic heart disease, and heart failure. Since patients with these backgrounds are at high risk for ischemic stroke, brain MRI was frequently performed to rule out stroke and to evaluate the ischemic status. The other inclusion criteria for the present analysis were: (1) data available on frailty status assessed using either of the two diagnostic criteria of frailty explained below, a modified version of the Cardiovascular Health Study (CHS) criteria (mCHS; Tamura et al., 2018) or Kihon Checklist (KCL), at baseline and at least one more time point till October 6, 2021; (2) the patients who were not frail at baseline (unaffected) by either criterion that met condition (1). Accordingly, data from 184 patients were eligible for inclusion. The number of patients who had both mCHS and KCL data at baseline was 182. For each of the mCHS and KCL, data from 137 and 154 patients unaffected at baseline were used, respectively. DM was diagnosed as previously described (Tamura et al., 2021b). The study protocol was approved by the ethics committee of the Tokyo Metropolitan Geriatric Hospital (R15-20, 19-03). Written informed consent was obtained from all patients. Patients with a history of brain neoplasm, neurosurgery, traumatic brain injury, psychiatric or neurological illness, or gross intracranial hemorrhage were excluded from this study.

### Assessment of frailty

Various diagnostic criteria for frailty have been proposed. The gold standard for diagnosing physical frailty is the CHS criteria, which is based on the abovementioned definition by Fried et al. (2001). Many modified versions of the CHS criteria have been developed, including the J-CHS, which is a modified version of the CHS criteria that is suitable for use in Japanese older adults (Satake et al., 2017a); the J-CHS was modified in 2020 (Satake and Arai, 2020). We have previously used the mCHS criteria that comprised almost the same items as the J-CHS and reported the prevalence of frailty in our outpatient clinic (Tamura et al., 2018).

Recently, the concept of frailty has been broadened to include comprehensive geriatric assessment, which includes the assessment of physical and cognitive function and social status. KCL defines criteria based on this broadened concept of frailty. High KCL scores have been associated with several CHS frailty phenotypes (Satake et al., 2016), mortality, disability (Satake et al., 2017b), and hospitalization (Koyama et al., 2022) in community-dwelling Japanese older adults.

In this study, the patients’ frailty status was determined using the mCHS and KCL. Patients with ≥3 out of 5 points on the mCHS were considered frail. However, based on recent revisions in the J-CHS (Satake and Arai, 2020), we revised the cutoff value of grip strength in men from 26 kg to 28 kg. The KCL is a multidomain questionnaire for evaluating frailty status, and patients with a score ≥8 were classified as frail (Satake et al., 2016). The Mini-Mental State Examination (MMSE) and the Japanese version of the Montreal Cognitive Assessment (MoCA-J) were used to evaluate cognitive function, and those with MMSE ≥ 24 and MoCA-J ≤ 25 were defined as suspected mild cognitive impairment (MCI) cases. The International Physical Activity Questionnaire was used to calculate the patients’ physical activity (metabolic equivalents × min/week), as described by Craig et al. (2003).

### MRI acquisition and DTI analysis

As performed in our previous study (Tamura et al., 2021b), MRI acquisition was carried out using a Discovery MR750w 3.0 MRI system with a 16-channel head coil (General Electric Healthcare, Milwaukee, WI, USA).

DTI acquisitions were executed using a single-shot spin-echo echoplanar imaging sequence with diffusion gradient encoding schemes consisting of 32 non-collinear directions with a *b*-value of 1,000 s/mm^2^ and one non-diffusion-weighted image with a *b*-value of 0 s/mm^2^, in the axial plane along the anterior-posterior commissure line. The sequence parameters were as follows: time of repetition/time of echo 17,000/95.7 ms; slices 66, without gaping; voxel size 1 × 1 × 2 mm^3^; matrix 256 × 256, field of view = 256 mm; number of excitations = 1).

DTI analysis was performed using the Oxford University FMRIB Software Library (Jenkinson et al., 2012). As shown in our previous report (Tamura et al., 2021b), we first corrected the eddy currents and movements in the DTI data of the patients using “eddy_correct.” We then created a brain mask by running “bet” on non-diffusion weighting images and fit the diffusion tensor model using “DTI fit.”

Two DTI indices, fractional anisotropy (FA) and mean diffusivity (MD), were calculated. FA has a range of 0–1, and low values indicate poor white matter tract integrity. In contrast, high MD values indicate poor integrity. Subsequently, we applied non-linear registration of FA or MD images onto the probabilistic brain atlas MNI152 to normalize them and extracted the mean FA skeletons of all patients, which represented and ran in the center of each tract. Finally, we projected each patient’s FA or MD values onto each skeleton of the tract.

In our previous study (Tamura et al., 2021b), we investigated the association between DTI parameters and sarcopenia using the following seven tracts as regions of interest (ROI): lATR, right ATR (rATR), forceps minor of the corpus callosum (FM), left IFOF (lIFOF), rIFOF, and left and right superior longitudinal fasciculus (SLF). This was because we hypothesized that executive and visuospatial dysfunction might be involved in the progression of sarcopenia and frailty, and it had been shown that these tracts might have some roles in these functions. Therefore, we selected these seven tracts as ROIs in this study.

### Statistical analyses

First, comparisons of DTI abnormalities between those who became frail during the follow-up period and those who did not were performed using the Mann–Whitney U test. Next, Cox proportional hazard models were used with the incidence of frailty according to each diagnostic criterion (mCHS and KCL) set as the objective variable and the following explanatory variables: FA or MD values plus age, sex, and body mass index (BMI) (Model 1); Model 1 plus hemoglobin A1c level (HbA1c), systolic blood pressure (sBP), and physical activity (Model 2); and Model 2 plus MMSE score (Model 3); BMI, physical activity and MMSE were significantly different in the Mann–Whitney U test in at least one of the frailty criteria. sBP is associated with deterioration of DTI markers in some tracts (Rosano et al., 2015; Tamura et al., 2021a). High HbA1c is shown to be associated with the incidence of frailty. Finally, we performed a stratified analysis of Model 3 based on the diagnostic status of DM. Estimated frailty-free survival curves were drawn by the Kaplan–Meier method and differences in survival between high MD and low MD groups in the lATR were evaluated with the log-rank test.

Statistical significance was set at *P* < 0.05. All analyses were performed using SPSS Version 20.0 (IBM Corp., Armonk, NY, USA).

## Results

### Baseline characteristics of the patients

The baseline characteristics of the selected patients, those who were not frail according to either the mCHS or KCL criteria at baseline and those with follow-up data of the frailty status, are shown in Table 1. The prevalence of diabetes, hypertension, and dyslipidemia at baseline were 62.5%, 73.9%, and 73.4%, respectively.

**Table 1:** Baseline clinical characteristics of the patients.

	**Total (*n* = 184)**	**Diabetes Mellitus (-) (*n* = 69)**	**Diabetes Mellitus (+) (*n* = 115)**	**p**
Age	77 [74–81]	76 [74–82]	77 [75–81]	0.437
Women (%)	64.1	71.0	60.0	0.132
BMI (kg/m^2^)	23.2 [21.1–25.7]	22.5 [20.5–25.5]	23.2 [21.1–25.7]	0.107
Hypertension (%)	73.9	75.4	73.0	0.729
Diabetes Mellitus (%)	62.5	0	100	–
Dyslipidemia (%)	73.4	66.7	77.4	0.111
Cardiovascular disease (%)	15.3	10.3	18.3	0.148
sBP (mmHg)	130 [119–140]	129 [116–139]	130 [121–140]	0.408
dBP (mmHg)	74 [67–82]	73 [67–82]	74 [68–82]	0.859
Alb (g/dL)	4.0 [3.9–4.2]	4.0 [3.8–4.2]	4.0 [3.9–4.2]	0.496
Hb (g/dL)	13.0 [12.4–13.8]	13.1 [12.5–14.0]	13.0 [12.3–13.8]	0.571
HbA1c (%)	6.5 [5.9–7.2]	5.9 [5.7–6.1]	7.0 [6.6–7.5]	**<0.001**
GA/HbA1c	2.73 [2.58–2.88]	2.69 [2.58–2.82]	2.81 [2.56–2.97]	0.051
TG (mg/dL)	115 [82–153]	112 [79–170]	116 [83–147]	0.634
LDL-C (mg/dL)	107 [89–125]	117 [96–139]	102 [87–120]	**<0.001**
HDL-C (mg/dL)	57 [48–69]	60 [51–71]	55 [46–66]	0.058
Cre (mg/dL)	0.80 [0.67–1.03]	0.77 [0.66–0.95]	0.81 [0.70–1.05]	0.174
eGFR (mL/min/1.73 m^2^)	58.2 [47.9–69.5]	60.2 [48.6–69.9]	56.6 [47.6–69.2]	0.434
MMSE	29 [27–29]	29 [28–29]	28 [26–29]	0.074
Suspected MCI (%)	75.5	71.0	78.3	0.268
EE (metabolic equivalents × min/week)	1386 [594–2772]	1386 [811–2354]	1386 [491–2777]	0.751
mCHS score	1 [0–2]	1 [0–2]	1 [0–2]	0.346
KCL score	5 [3–7]	5 [3–7]	5 [3–7]	0.964

Data are presented as median [25–75 percentile]. BMI, body mass index; sBP, systolic blood pressure; dBP, diastolic blood pressure; Alb, albumin level; Hb, hemoglobin level; GA, Glucoalbumin level; HbA1c, hemoglobin A1c level; TG, triglyceride level; LDL-C, low-density lipoprotein cholesterol level; HDL-C, high-density lipoprotein cholesterol level; Cre, creatinine level; eGFR, estimated glomerular filtration rate; MMSE, Mini-Mental State Examination score; MCI, mild cognitive impairment; EE, energy expenditure; mCHS, modified Cardiovascular Health Study criteria; KCL, Kihon Checklist. MCI was defined as MMSE ≥ 24 and MoCA-J ≤ 25. Bold values indicate statistical significance *p* < 0.05.

### Comparisons of DTI abnormalities at baseline between those who did and did not develop frailty

Among the 137 patients who were not diagnosed as frail at baseline according to the mCHS criteria, 46 (34%) developed frailty during a median follow-up period of 3.0 years. Similarly, among 154 patients who were not diagnosed as frail according to the KCL criteria, 48 (31%) developed frailty during the median follow-up period of 3.0 years. Among the 107 patients who were not diagnosed as frail according to both criteria and whose subsequent frailty status according to both criteria were available, 12 (11%) developed frailty according to the mCHS criteria alone, 10 (9%) developed frailty according to the KCL criteria alone, and 19 (18%) developed frailty according to both criteria.

The results of the Mann–Whitney U test are shown in Table 2. Among the patients without frailty at baseline according to mCHS criteria, those who developed frailty showed lower baseline FA values in many tracts, including the FA in the rATR, FM, lIFOF, and bilateral SLF, and higher MD values in all seven tested tracts, compared with patients who did not develop frailty. Similarly, those who developed frailty according to the KCL criteria showed significantly lower baseline FA and higher MD values in the bilateral ATR than those who did not develop frailty. In both diagnostic criteria, the tracts with the most significant differences were the MD values in the rATR, with values of 8.06 × 10^−4^ vs. 7.80 × 10^−4^, *p* = 0.001 and 8.06 × 10^−4^ vs. 7.81 × 10^−4^, *p* = 0.001 (mCHS and KCL, respectively).

**Table 2:** Differences in FA and MD values in the tested tracts at baseline between the patients with and without frailty as defined using the mCHS and KCL.

**Tracts**		**mCHS (*n* = 137)**		**KCL (*n* = 154)**	
		**Frailty (+) (*n* = 46)**	**Frailty (-) (*n* = 91)**	**Frailty (+) (*n* = 48)**	**Frailty (-) (*n* = 106)**
lATR	FA	0.529 (0.501–0.546)	0.533 (0.516–0.557)	**0.524 (0.503–0.542)**	0.538 (0.518–0.559)**
	MD(×10^−4^)	**7.83 (7.43–8.16)**	7.45 (7.16–7.87)**	**7.69 (7.29–8.21)**	7.44 (7.18–7.77)**
rATR	FA	**0.501 (0.479–0.520)**	0.510 (0.492–0.529)*	**0.502 (0.487–0.521)**	0.511 (0.496–0.530)*
	MD(×10^−4^)	**8.06 (7.93–8.66)**	7.80 (7.51–8.19)**	**8.06 (7.76–8.39)**	7.81 (7.55–8.09)**
FM	FA	**0.551 (0.536–0.568)**	0.561 (0.545–0.581)*	0.561 (0.538–0.577)	0.564 (0.548–0.584)
	MD(×10^−4^)	**8.14 (7.89–8.36)**	7.94 (7.68–8.20)**	7.92 (7.65–8.28)	7.96 (7.73–8.21)
lIFOF	FA	**0.527 (0.506–0.547)**	0.539 (0.516–0.562)*	0.538 (0.507–0.556)	0.540 (0.520–0.563)
	MD(×10^−4^)	**8.24 (7.94–8.47)**	8.04 (7.77–8.29)*	8.04 (7.84–8.39)	8.02 (7.80–8.33)
rIFOF	FA	0.524 (0.508–0.538)	0.535 (0.511–0.554)	0.525 (0.508–0.556)	0.540 (0.513–0.553)
	MD(×10^−4^)	**8.22 (8.02–8.42)**	7.99 (7.76–8.35)**	8.10 (7.81–8.35)	8.02 (7.77–8.37)
lSLF	FA	**0.506 (0.482–0.528)**	0.521 (0.503–0.537)*	0.520 (0.501–0.540)	0.522 (0.505–0.537)
	MD(×10^−4^)	**7.63 (7.40–7.93)**	7.52 (7.27–7.71)*	7.49 (7.27–7.72)	7.45 (7.25–0.770)
rSLF	FA	**0.511 (0.481–0.533)**	0.527 (0.501–0.540)*	0.523 (0.505–0.541)	0.527 (0.501–0.540)
	MD(×10^−4^)	**7.73 (7.36–8.00)**	7.52 (7.27–7.69)**	7.55 (7.28–7.77)	7.51 (7.24–0.770)

FA, fractional anisotropy; MD, mean diffusivity; mCHS, modified Cardiovascular Health Study criteria; KCL, Kihon Checklist; lATR, left anterior thalamic radiation; rATR, right anterior thalamic radiation; FM, forceps minor, lIFOF, left inferior fronto-occipital fasciculus; rIFOF, right inferior fronto-occipital fasciculus; lSLF, left superior longitudinal fasciculus; rSLF, right superior longitudinal fasciculus; Data are presented as median (25–75 percentile). *p < 0.05, **p < 0.01 vs. Frailty(+). Bold values indicate statistical significance *p* < 0.05.

### Cox proportional hazard models for the incidence of frailty

The results are shown in Table 3A (mCHS-defined frailty) and Table 3B (KCL-defined frailty). The MD value in the lATR showed a significant association with the incidence of frailty according to the mCHS criteria in Models 1, 2, and 3. Similarly, the MD values in the bilateral ATR were significantly associated with the incidence of frailty according to the KCL criteria.

**Table 3A:** Cox proportional hazard models of the DTI values for the incidence of frailty according to the mCHS (*n* = 137).

		**Model 1**		**Model 2**		**Model 3**	
**Tract**		**HR (95%CI)**	**p**	**HR (95%CI)**	**p**	**HR (95%CI)**	**p**
lATR	FA	1.481 (0.514–4.268)	0.467	1.522 (0.505–4.583)	0.456	1.460 (0.492–4.331)	0.496
	MD	**2.137 (1.247–3.661)**	**0.006**	**2.161 (1.201–3.887)**	**0.010**	**2.029 (1.134–3.631)**	**0.017**
rATR	FA	1.226 (0.385–3.902)	0.730	1.298 (0.412–4.091)	0.656	1.365 (0.426–4.373)	0.600
	MD	1.462 (0.946–2.260)	0.087	1.551 (0.954–2.522)	0.077	1.615 (0.985–2.649)	0.058
FM	FA	1.173 (0.359–3.930)	0.791	1.012 (0.314–3.262)	0.984	1.345 (0.420–4.308)	0.618
	MD	1.614 (0.741–3.515)	0.229	1.395 (0.626–3.109)	0.415	1.611 (0.753–3.447)	0.219
lIFOF	FA	1.252 (0.456–3.438)	0.662	1.463 (0.511–4.188)	0.479	1.614 (0.565–4.611)	0.372
	MD	1.040 (0.495–2.184)	0.918	1.163 (0.535–2.528)	0.704	1.295 (0.595–2.819)	0.514
rIFOF	FA	0.861 (0.364–2.037)	0.733	1.087 (0.445–2.657)	0.855	1.206 (0.475–3.061)	0.694
	MD	0.982 (0.733–1.316)	0.904	1.066 (0.790–1.438)	0.677	1.122 (0.826–1.524)	0.462
lSLF	FA	1.037 (0.451–2.383)	0.932	1.180 (0.526–2.645)	0.688	1.358 (0.590–3.129)	0.472
	MD	0.879 (0.503–1.536)	0.650	0.892 (0.497–1.602)	0.703	0.994 (0.552–1.792)	0.985
rSLF	FA	1.063 (0.544–2.078)	0.859	1.161 (0.584–2.308)	0.671	1.262 (0.625–2.548)	0.516
	MD	1.019 (0.568–1.826)	0.950	1.067 (0.587–1.939)	0.832	1.170 (0.637–2.151)	0.612

Model 1: Adjusted for age, sex, and body mass index at baseline. Model 2: Model 1 plus systolic blood pressure, hemoglobin A1c level, and physical activity at baseline. Model 3: Model 2 plus MMSE score at baseline. DTI, diffusion tensor imaging; mCHS, modified Cardiovascular Health Study criteria; FA, fractional anisotropy; MD, mean diffusivity; lATR, left anterior thalamic radiation; rATR, right anterior thalamic radiation; FM, forceps minor, lIFOF, left inferior fronto-occipital fasciculus; rIFOF, right inferior fronto-occipital fasciculus; lSLF, left superior longitudinal fasciculus; rSLF, right superior longitudinal fasciculus; MMSE, Mini-Mental State Examination score; HR, hazard ratio; CI, confidence interval. Hazard ratios are shown per 0.1 decrease for FA and per 1 × 10^−4^ increase for MD. Bold values indicate statistical significance *p* < 0.05.

**Table 3B:** Cox proportional hazard models of the DTI values for the incidence of frailty according to the KCL criteria (*n* = 154).

		**Model 1**		**Model 2**		**Model 3**	
**Tract**		**HR (95%CI)**	**p**	**HR (95%CI)**	**p**	**HR (95%CI)**	**p**
lATR	FA	**4.834 (1.862–12.55)**	**0.001**	**4.576 (1.630–12.85)**	**0.004**	**4.259 (1.561–11.62)**	**0.005**
	MD	**1.918 (1.250–2.942)**	**0.003**	**1.770 (1.143–2.740)**	**0.010**	**1.748 (1.111–2.752)**	**0.016**
rATR	FA	**5.301 (1.486–18.91)**	**0.010**	**3.921 (1.125–13.66)**	**0.032**	**3.959 (1.114–14.07)**	**0.033**
	MD	**2.138 (1.390–3.289)**	**0.001**	**1.893 (1.239–2.893)**	**0.003**	**1.938 (1.252–2.999)**	**0.003**
FM	FA	2.899 (0.929–8.980)	0.067	2.637 (0.780–8.912)	0.119	**3.589 (1.047–12.30)**	**0.042**
	MD	1.267 (0.574–2.800)	0.558	1.175 (0.510–2.711)	0.705	1.536 (0.655–3.601)	0.324
lIFOF	FA	2.489 (0.967–6.404)	0.059	2.485 (0.909–6.799)	0.076	**2.844 (1.039–7.782)**	**0.042**
	MD	1.214 (0.563–2.618)	0.620	1.306 (0.615–2.774)	0.488	1.476 (0.677–3.216)	0.328
rIFOF	FA	**2.927 (1.118–7.664)**	**0.029**	**2.919 (1.086–7.847)**	**0.034**	**3.845 (1.355–10.91)**	**0.011**
	MD	**1.489 (1.018–2.178)**	**0.040**	1.433 (0.996–2.063)	0.053	1.536 (0.655–3.601)	0.324
lSLF	FA	1.591 (0.613–4.133)	0.340	1.753 (0.658–4.672)	0.262	2.038 (0.739–5.621)	0.169
	MD	1.771 (0.930–3.371)	0.082	**1.929 (1.029–3.617)**	**0.040**	**2.350 (1.229–4.494)**	**0.010**
rSLF	FA	1.362 (0.599–3.100)	0.461	1.556 (0.668–3.622)	0.305	1.675 (0.714–3.926)	0.236
	MD	1.450 (0.801–2.627)	0.220	1.799 (0.963–3.360)	0.066	**2.034 (1.075–3.847)**	**0.029**

Model 1: Adjusted for age, sex, and body mass index at baseline. Model 2: Model 1 plus systolic blood pressure, hemoglobin A1c level, and physical activity at baseline. Model 3: Model 2 plus MMSE score at baseline. DTI, diffusion tensor imaging; KCL, Kihon Checklist; FA, fractional anisotropy; MD, mean diffusivity; lATR, left anterior thalamic radiation; rATR, right anterior thalamic radiation; FM, forceps minor, lIFOF, left inferior fronto-occipital fasciculus; rIFOF, right inferior fronto-occipital fasciculus; lSLF, left superior longitudinal fasciculus; rSLF, right superior longitudinal fasciculus; MMSE, Mini-Mental State Examination score; HR, hazard ratio; CI, confidence interval. Hazard ratios are shown per 0.1 decrease for FA and per 1 × 10^−4^ increase for MD. Bold values indicate statistical significance *p* < 0.05.

Additionally, the DTI values of the following tracts were associated with the incidence of KCL-defined frailty: the FA in bilateral ATR and rIFOF showed statistically significant associations, while that in the FM and lIFOF showed weak associations. For MD, bilateral SLF also showed association trends. Specifically, the hazard ratios of KCL-defined frailty incidence were 4.26 when the FA of the lATR dropped by 0.1 and 1.75 when the MD of the tract rose by 1 × 10^−4^. However, for mCHS-defined frailty, no other tested tracts showed a significant association. In the stratified analysis (Model 3) based on the diagnostic status of DM, results similar to those of the total sample were observed among patients with DM. Contrarily, among those without DM, the association between DTI changes and incidence of frailty was found in none of the tested tracts and with none of the frailty definitions (Table 4).

**Table 4:** Cox regression analysis of the DTI values for the incidence of frailty stratified according to diabetes mellitus status.

		**mCHS (*n* = 137)**				**KCL (*n* = 154)**			
		**Diabetes Mellitus (-)** (*n* = 48)		**Diabetes Mellitus (+) (*n* = 89)**		**Diabetes Mellitus (-) (*n* = 58)**		**Diabetes Mellitus (+) (*n* = 96)**	
**Tract**		**HR (95%CI)**	**p**	**HR (95%CI)**	**p**	**HR (95%CI)**	**p**	**HR (95%CI)**	**p**
lATR	FA	0.159 (0.008–3.321)	0.231	2.084 (0.622–6.989)	0.234	13.71 (0.815–230.5)	0.069	**4.208 (1.217–14.55)**	**0.023**
	MD	1.468 (0.329–6.554)	0.615	**2.231 (1.132–4.396)**	**0.020**	2.684 (0.630–11.43)	0.182	**1.828 (1.098–3.043)**	**0.020**
rATR	FA	0.266 (0.007–10.55)	0.481	1.612 (0.376–6.908)	0.520	3.142 (0.158–62.40)	0.453	**5.830 (1.239–27.44)**	**0.026**
	MD	2.270 (0.481–10.71)	0.300	1.622 (0.907–2.902)	0.103	1.926 (0.485–7.650)	0.352	**2.331 (1.421–3.824)**	**0.001**
FM	FA	0.079 (0.003–2.194)	0.134	3.085 (0.592–16.08)	0.181	10.92 (0.802–148.7)	0.073	3.538 (0.792–15.81)	0.098
	MD	0.343 (0.030–3.946)	0.391	2.897 (0.960–8.738)	0.059	5.457 (0.960–31.03)	0.056	1.190 (0.434–3.265)	0.736
lIFOF	FA	0.394 (0.029–5.315)	0.483	3.200 (0.830–12.34)	0.091	1.717(0.157–18.83)	0.658	**3.926 (1.215–12.69)**	**0.022**
	MD	0.078 (0.003–2.033)	0.125	2.334 (0.843–6.460)	0.103	2.132 (0.359–12.66)	0.405	1.522 (0.601–3.849)	0.375
rIFOF	FA	0.362 (0.022–5.995)	0.478	1.559 (0.505–4.808)	0.440	3.188 (0.296–34.33)	0.339	**5.033 (1.439–17.61)**	**0.011**
	MD	0.076 (0.005–1.183)	0.066	1.163 (0.826–1.637)	0.387	1.917 (0.320–11.48)	0.476	**1.713 (1.153–2.544)**	**0.008**
lSLF	FA	0.386 (0.052–2.872)	0.353	2.547 (0.742–8.740)	0.137	1.650 (0.294–9.268)	0.570	2.720 (0.669–11.06)	0.162
	MD	0.372 (0.064–2.159)	0.270	1.192 (0.566–2.513)	0.644	2.077 (0.645–6.682)	0.220	**2.927 (1.283–6.675)**	**0.011**
rSLF	FA	0.527 (0.087–3.209)	0.487	1.802 (0.701–4.633)	0.221	1.674 (0.380–7.374)	0.496	2.010 (0.626–6.453)	0.241
	MD	0.696 (0.180–2.695)	0.600	1.603 (0.667–3.852)	0.291	1.583 (0.570–4.287)	0.386	**3.325 (1.229–8.993)**	**0.018**

Adjusted for age, sex, body mass index, systolic blood pressure, hemoglobin A1c level, physical activity, and MMSE score at baseline (Model 3). DTI, diffusion tensor imaging; FA, fractional anisotropy; MD, mean diffusivity; mCHS, modified Cardiovascular Health Study criteria; KCL, Kihon Checklist; lATR, left anterior thalamic radiation; rATR, right anterior thalamic radiation; FM, forceps minor, lIFOF, left inferior fronto-occipital fasciculus; rIFOF, right inferior fronto-occipital fasciculus; lSLF, left superior longitudinal fasciculus; rSLF, right superior longitudinal fasciculus; MMSE, Mini-Mental State Examination score; HR, hazard ratio; CI, confidence interval. Hazard ratios are shown per 0.1 decrease for FA and per 1 × 10^−4^ increase for MD. Bold values indicate statistical significance *p* < 0.05.

**Figure 1:**
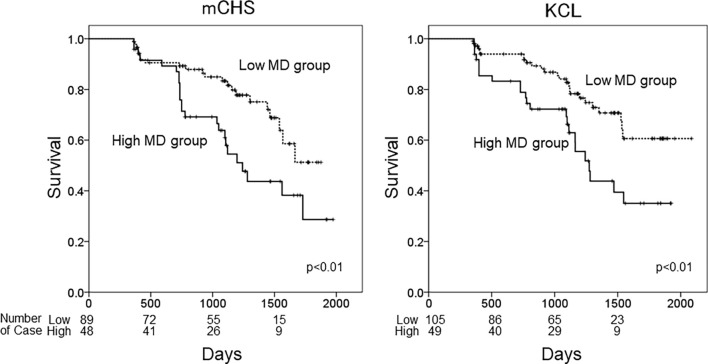
Kaplan–Meiercurves of frailty-free survival. The patients were categorized into two groups based on frailty defined using the mCHS or KCL and based on the cutoff MD values of the left ATR. High MD group: MD ≥ 7.8 × 10^−4^, low MD group: MD < 7.8 × 10^−4^. mCHS, modified Cardiovascular Health Study criteria; KCL, Kihon Checklist; lATR, left anterior thalamic radiation; MD, mean diffusivity; rATR, right anterior thalamic radiation.

Finally, we performed Cox regression analysis by adding the history of symptomatic stroke as a confounding factor. The effects of changes in DTI values in some tracts (rATR, FM, and lIFOF) on the incidence of KCL-defined frailty were slightly attenuated in all patients and those with DM. However, the associations of lATR values on the incidence of both mCHS-defined and KCL-defined frailty remained significant even after adding symptomatic stroke as an explanatory variable. Figure 1 shows the Kaplan–Meier curve of the frailty-free survival rate in patients with high MD (≥7.8 × 10^−4^) and low MD (<7.8 × 10^−4^) in the lATR. In both frailty criteria, the high MD group showed a significantly higher rate of frailty incidence than the low MD group (*p* < 0.01 in both; log-rank test).

## Discussion

In this study, we prospectively investigated the association between white matter changes evaluated using DTI and the incidence of frailty; abnormalities in some tracts, especially the ATR, were found to be involved in the association.

The strength of this study is that, unlike previous studies that included healthy community-dwelling older adults, we used the data of a substantial number of outpatients (>180), most of whom had cardiometabolic diseases, accumulation of which is a strong risk for frailty as well as for white matter deterioration. In our recent cross-sectional study, some early markers for atherosclerosis were associated with changes in DTI indices (Tamura et al., 2021a). It is important to understand how changes in white matter integrity affect the frailty status of these high-risk patients. Furthermore, we evaluated frailty status using the mCHS and KCL. Recently, it has been emphasized that frailty should not just be evaluated using a physical frailty scale, but scales should encompass broad aspects such as cognitive function and social activity since these factors correlate with each other (Ma et al., 2018). The KCL incorporates various domains such as cognitive dysfunction, malnutrition, social isolation, and depression and is associated not only with the number of CHS frailty phenotypes but also with nutritional state, cognitive function, and depressive mood (Satake et al., 2016), which could be a reason for its strong association with DTI changes.

Herein, we found that alterations in DTI values in the ATR at baseline had the strongest influence on the incidence of frailty. Only a few studies have investigated the association between frailty and abnormalities in white matter integrity assessed using DTI. A cross-sectional study (Avila-Funes et al., 2017) and a longitudinal study (Maltais et al., 2020) investigated the associations between lesion localization and outcome; both studies demonstrated that lesions of the anterior limb of the internal capsule were among those responsible for the presence or worsening of frailty. The method of classifying white matter tracts in our study differed from those of the studies mentioned above as we did not directly verify the anterior limb of the internal capsule. Nevertheless, as ATR fibers run through the anterior limb of the internal capsule, our results are partially comparable with their results. We found that changes in MD in the lATR influenced both mCHS- and KCL-defined frailty. The ATR is involved in gait speed and executive function (Poole et al., 2018), indicating that this region is crucial for maintaining functional and cognitive ability. However, since the association between MD values and the incidence of frailty was observed even after adjusting for physical activity and cognitive function, DTI changes could induce frailty *via* an unknown pathway independent of these dysfunctions.

Conversely, we found an association between FA change and outcomes only in KCL-defined frailty and not in mCHS-defined frailty, indicating that changes in MD values in the ATR were more strongly associated with CHS-defined frailty than with changes in FA. This is similar to the results obtained by Maltais et al. (2020) probably because the FA values in the crossing-fiber regions are underestimated (Jbabdi et al., 2010). However, it is unclear why the difference was significant only in KCL- and not in mCHS-defined frailty.

In other tracts, the results of our study were mostly compatible with those of Maltais et al. (2020). Specifically, both studies showed a significant association between the alteration of MD in the left SLF and worsening of frailty, although the definition of frailty used was different from that in the mCHS and KCL. As multiple reports have shown that the integrity of the SLF is associated with gait speed (Rosario et al., 2016; Poole et al., 2018), it is natural that abnormalities in this region can lead to frailty. However, we observed only a weak effect of the DTI values of FM on the incidence of frailty, similar to Maltais et al. (2020), who showed no association between FA or MD values of the body or genu of the corpus callosum (CC) and a worsening frailty phenotype. The CC connects the bilateral hemispheres and plays multiple roles. The FM is a group of tracts that extend from the anterior CC (genu) to the cortex of the frontal lobes (Mamiya et al., 2018). It has been indicated that DTI alterations in the FM and the ATR are associated with impaired executive function in young bilingual adults (Mamiya et al., 2018) and that alterations in the genu of the CC are associated with impaired executive function (Zheng et al., 2014) and poor gait performance (de Laat et al., 2011); however, the total effect of lesions in FM on the progression of frailty might be somewhat smaller than those in the ATR or SLF.

We found that low FA and high MD values in the rIFOF were associated with a high incidence of KCL-defined frailty, although the difference was not statistically significant (Table 2). The IFOF connects the frontal and occipital lobes (Jou et al., 2011) and is involved in visuospatial cognition (Voineskos et al., 2012), together with superior FOF; Maltais et al. (2020) showed that the MD values of this tract were associated with frailty incidence. Our previous cross-sectional study demonstrated that the abnormalities in the lATR and rIFOF are associated with the prevalence of sarcopenia, especially in patients with DM (Tamura et al., 2021b). These results suggest that alterations in the integrity of the IFOF might induce frailty *via* the impairment of muscle function. Further studies are needed to clarify how changes in the integrity of specific tracts can lead to frailty.

In the stratified analysis, we found associations between the changes in DTI values and the incidence of frailty, specifically in patients with DM and not in those without DM. This could be because patients with DM may have a concurrent etiological background for both white matter changes and the incidence of frailty. Insulin resistance and oxidative stress could be candidate factors; however, we could not evaluate these factors in this study. Individuals with high insulin resistance reportedly show alterations in DTI values in broad white matter tracts (Ryu et al., 2014). Contrarily, insulin resistance is strongly related to and forms a vicious cycle of sarcopenia and sarcopenic obesity (Cleasby et al., 2016), which are key factors for frailty progression. Nonetheless, in those without DM, other physical factors besides white matter changes could play a more important role in developing frailty.

This study had several limitations. First, the follow-up rate was low. We could not evaluate the frailty status of approximately half of the patients at 2 years. The incidence of frailty might have been higher if those who dropped out had been analyzed. Second, although the general rule was to perform the follow-up assessment annually, some patients could not be evaluated every year. This may have resulted in an overestimation of the period until the incidence of frailty. Third, we did not control for multiple comparisons in multivariate analyses and this might lead to overestimations of some of the results. Fourth, we did not evaluate changes in DTI values. The causative effect of the deterioration of white matter integrity over time on the incidence of frailty could not be clarified. Finally, this study was conducted at a single Japanese facility. Multicenter studies are needed to extrapolate our results to a larger population.

In conclusion, changes in various white matter tracts are associated with the incidence of frailty in older adults, especially those with DM. Changes in DTI values in the ATR were most closely related to the incidence of frailty, and it can be used as an early predictive marker of frailty. Future studies are needed to determine whether diet, exercise, and pharmacologic interventions can improve DTI abnormalities or prevent the progression of frailty in patients at high risk for frailty, as revealed by these DTIs.

## Data Availability Statement

The datasets presented in this article are not readily available due to participants’ confidentiality. Requests to access the datasets should be directed to Yoshiaki Tamura: tamurayo@tmghig.jp.

## Ethics Statement

The studies involving human participants were reviewed and approved by the ethics committee of the Tokyo Metropolitan Geriatric Hospital. The patients/participants provided their written informed consent to participate in this study.

## Author Contributions

YT, JI, and AA designed the study, analyzed the data, and wrote the draft of the manuscript. KS contributed to DTI data collection. YM, FY, RK, KO, KT, and YC contributed to clinical data collection, analysis, and interpretation of data. AT contributed to data interpretation and critically reviewed the manuscript. All authors contributed to the article and approved the submitted version.

## Conflict of Interest

AA has received speaker honoraria from Merck Sharp & Dohme, Sumitomo Parma Co. Ltd., Tanabe Mitsubishi Pharma Corporation, Ono Pharmaceutical Co. Ltd., Takeda Pharmaceutical Co. Ltd., and Novo Nordisk Pharma Ltd. The remaining authors declare that the research was conducted in the absence of any commercial or financial relationships that could be construed as a potential conflict of interest.

## Publisher’s Note

All claims expressed in this article are solely those of the authors and do not necessarily represent those of their affiliated organizations, or those of the publisher, the editors and the reviewers. Any product that may be evaluated in this article, or claim that may be made by its manufacturer, is not guaranteed or endorsed by the publisher.
